# The health cost of urbanization: identification and ranking of influencing factors of class A and B infectious diseases based on machine learning

**DOI:** 10.3389/fpubh.2025.1616841

**Published:** 2025-12-03

**Authors:** Zhiqing Wang, Boyi Zhu, Liuyu Wu

**Affiliations:** 1 School of Public Policy and Management, Tsinghua University, Beijing, China; 2 Southwest Jiaotong University, Chengdu, China; 3 School of Accounting, Southwestern University of Finance and Economics, Chengdu, China

**Keywords:** public health, infectious diseases, urbanization, machine learning, influencing factors

## Abstract

**Aims:**

With the rapid advancement of urbanization, an increasing number of people are congregating in urban areas, leading to a higher density of economic activities. This may not only accelerate the spread of infectious diseases but also result in pollutants that harm residents’ health. Nevertheless, improvements in infrastructure and healthcare services, coupled with heightened awareness of personal protection among residents, can effectively mitigate the spread of infectious diseases. The incidence rates of Class A and B infectious diseases serve as critical indicators of public health status. This study seeks to identify and prioritize the key factors influencing public health during the process of rapid urbanization, thereby providing a scientific basis for decision-making aimed at enhancing residents’ living environments and addressing existing gaps in public health systems.

**Methods:**

Using provincial-level data from mainland China (2008–2022), this study systematically applied multiple machine learning methods, including Random Forest, Gradient Boosting Tree, and XGBoost, to evaluate the impacts of over 10 indicators across economic, demographic, land use, and social dimensions on the Incidence Rate of Class A and B Infectious Diseases per 100,000 Population.

**Results:**

(1) Social factors account for 46.7%, constituting the most significant determinant, succeeded by land use, population, and economic dimensions. (2) Public transportation, urban water supply coverage, healthcare expenditure, and the spatial distribution of healthcare resources exert direct effects on residents’ health outcomes and the accessibility of public health services. (3) Regarding land use, effective urban planning—reflected in indicators such as the green coverage rate of built-up areas and the per capita area of paved roads—plays a crucial role in promoting public health, accounting for 17.4% on average, whereas inadequate land-use management often precipitates health risks. (4) Population dynamics, encompassing demographic restructuring, agglomeration, and education levels, simultaneously generate advantages (e.g., improved efficiency in health service delivery) and challenges (e.g., heightened vulnerability to infectious disease transmission). (5) Economic factors, including industrial pollution control (ratio of completed investment in industrial pollution control to GDP), industrial upgrading (ratio of tertiary to secondary industry value-added), international trade (foreign trade dependence), and income levels (per capita disposable income of urban residents), manifest dual effects: advancing health improvements while engendering environmental degradation and cross-border health risks. (6) The health implications of rapid urbanization display regional disparity: social factors (60.2%) predominate in eastern China, economic factors (63.2%) in central China, and land use factors (54.2%) in western regions.

**Conclusion:**

During rapid urbanization, governments must prioritize timely enhancements to public health services, rational land use planning, and protection of vulnerable populations. Emphasizing the quality of economic development and fostering synergies between industrial upgrading and environmental governance will improve public health outcomes.

## 1 Introduction

With the rapid acceleration of global urbanization, human settlement patterns are experiencing an unprecedented structural transformation. According to statistics, approximately 55% of the global population currently resides in urban areas, and this proportion is projected to increase to 68% by 2050 (1). This process not only reshapes urban spatial configurations but also profoundly transforms residents’ living environments. On one hand, it leads to significant improvements in material living conditions, such as optimized dietary structures, enhanced housing quality, and improved transportation networks. On the other hand, it has a substantial impact on residents’ physical and mental health (1–3). Public health levels are crucial not only for individual development and family well-being but also for influencing regional economic efficiency and social governance through the mechanism of human capital accumulation (4, 5). However, urbanization and public health are not inherently synchronized. According to World Bank data, in the 21st century, the growth rate of the urban population in middle-income and low-income countries has significantly exceeded the global average. Despite this rapid urbanization, these regions have also experienced higher incidences of infectious diseases, revealing a structural imbalance between economic development and social governance. To effectively curb the spread of infectious diseases, enhance the public health system, and harmonize the relationship between economic development and public health, it is crucial to systematically analyze the determinants of public health and establish a multi-dimensional indicator framework that integrates economic development, demographic changes, land use patterns, and social infrastructure. This framework will provide a robust scientific foundation for decision-making aimed at creating healthy and livable cities, ultimately fostering a dynamic equilibrium between urban and individual development.

Urbanization exerts a dual and complex influence on public health. On the one hand, it reflects not only the rising proportion of urban populations but also advancements in infrastructure and the agglomeration of high-end resources, all of which contribute positively to improving public health conditions. On the other hand, the urbanization process is often accompanied by accelerated industrialization, transformations in residential and lifestyle patterns, and increased population density—factors that can amplify the spread of infectious diseases and exacerbate environmental pollution. These developments, in turn, pose significant threats to population health (2, 6–10).

Particularly during periods of rapid urbanization, insufficient or lagging infrastructure development and uncoordinated urban planning can lead to an uneven distribution of healthcare resources and facilitate the accelerated transmission of infectious diseases (11–16). However, existing studies on the determinants of public health often concentrate on single or narrowly defined indicators (17, 18), limiting their capacity to holistically assess the broad and multifaceted impacts of urbanization on health outcomes.

Moreover, conventional research methodologies frequently exhibit significant limitations in capturing the full scope of urbanization-related variables (19–22), with a restricted range of influencing factors considered. This methodological constraint hinders a comprehensive understanding of the diversity and complexity inherent in the urbanization–health nexus. In reality, the relationship between urbanization and public health is highly dynamic and context-dependent, with dominant influencing factors varying significantly across different stages of urban development (23).

**Figure 1:**
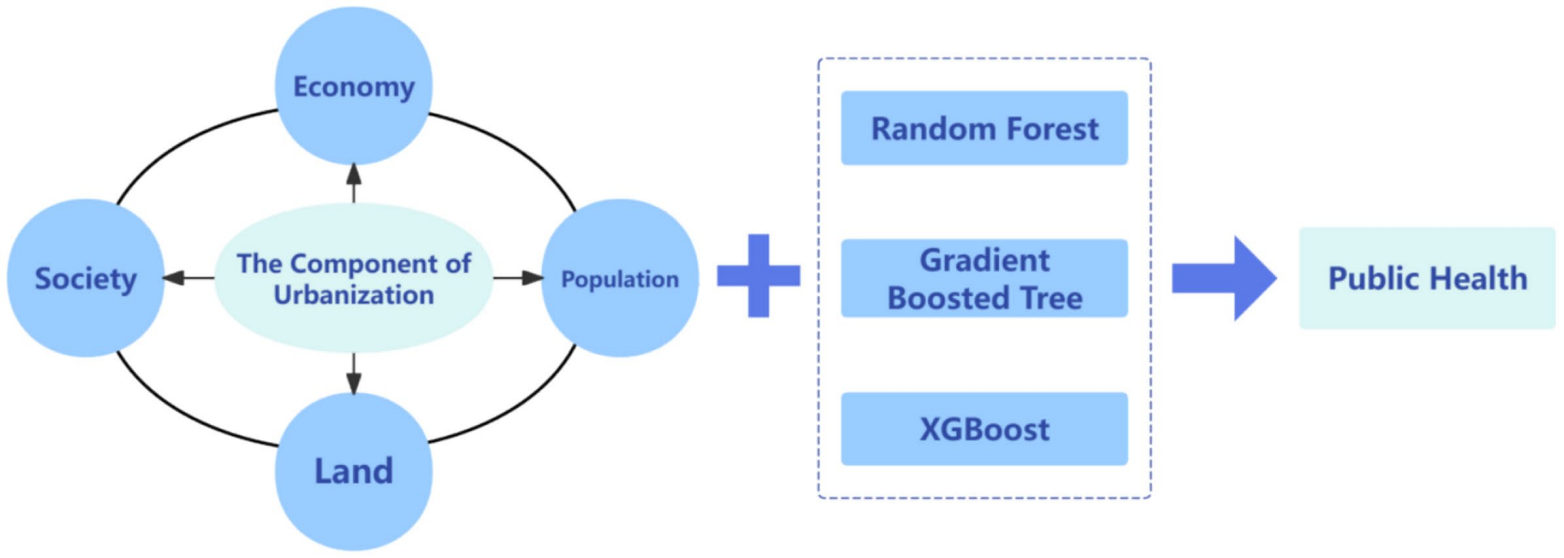
Research dimensions and research methods.

In contrast to traditional statistical methods, machine learning does not presuppose a functional form, thereby circumventing issues of functional misspecification (24). For instance, while public health is closely related to economic development levels and demographic factors, it is impractical to predefine an explicit functional relationship. Leveraging nonparametric estimation techniques, machine learning offers greater flexibility by identifying and retaining the most salient features from a vast array of variables without manual selection, thus partially mitigating the curse of dimensionality (25). A case in point is the multifactorial nature of public health, where pinpointing dominant influencing factors poses both a critical and pragmatic challenge. Furthermore, machine learning exhibits robust generalization capabilities, making it well-suited for predictive scenarios (26). These methods mitigate overfitting through cross-validation techniques and balance the trade-off between “predictive performance” and “generalization ability” through regularization strategies, as seen in algorithms such as Random Forest. Machine learning models like Random Forest require minimal hyperparameter tuning and are less dependent on prior assumptions about the data (27). To address the limitations of existing research, this study leverages provincial panel data from mainland China spanning 2008 to 2022. It constructs a system of over 10 indicators across four key dimensions—economic development, population change, land use patterns, and social infrastructure—and applies machine learning algorithms, including Random Forest, Gradient Boosting Trees (GDBT), and XGBoost, to quantitatively analyze the relative contributions of these dimensions to the incidence rates of class A and B statutory infectious diseases. This approach not only uncovers the complex, multifactorial causal mechanisms underlying the incidence rates of these infectious diseases during the urbanization process but also provides an evidence-based foundation for optimizing disease prevention and control policies. What’s more, Random Forest, GDBT, and XGBoost are robust (28), are adopted to represent both Bagging and Boosting families in ensemble learning. These three models have demonstrated excellent stability, interpretability, and generalization performance in empirical studies involving limited-sample socioeconomic and health data (27, 29). These findings offer valuable insights for both public health theory and practice, highlighting critical factors influencing disease dynamics in the context of urban development. Figure 1 shows the research dimensions and research methods.

## 2 Materials and methods

### 2.1 Data source

This study selects data from 30 provincial-level units on the mainland of China, covering the period from 2008 to 2022. Hong Kong, Macau, Taiwan, and Tibet are excluded from the research scope due to significant data gaps. Based on relevant literature (20, 40), the input variables are categorized into 16 variables in four major factors, namely, demographic, economic, land and social factors, and the data sources are all from the National Bureau of Statistics as well as provincial statistical yearbooks, while the output variable is the incidence rate of class A and B infectious diseases per 100,000 population, and the data source is the China Health Statistical Yearbook. The Total Dependency Ratio is not published for 2020. According to its definition, this study imputes the 2020 value based on census data, using the ratio of the non-working-age population (0–14 and 65+) to the working-age population (15–64). See Table 1 for descriptive statistics on the classification of variable categories.

**Table 1:** Descriptive statistical characteristics of variables.

Type	Variables	*N*	Mean	Std	Min	Max
Output variable	Incidence rate of class A and B infectious diseases per 100,000 population	450	240.13	99.63	74.39	738.19
Dem1	Total dependency ratio	450	38.08	7.453	19.30	57.79
Dem2	Urbanization rate	450	57.71	12.994	29.11	89.60
Dem3	Illiteracy rate	450	5.10	3.066	0.79	17.78
Dem4	Urban population density	450	2889.06	1169.59	649.00	5967.00
Eco1	Ratio of tertiary industry value-added to secondary industry value-added	450	1.29	0.722	0.53	5.24
Eco2	Foreign trade dependence	450	0.28	0.307	0.01	1.60
Eco3	*Per Capita* disposable income of urban residents	450	30504.56	13393.60	11413.00	84034.00
Eco4	Ratio of completed investment in industrial pollution control to GDP	450	0.12	0.125	0.00	1.10
Land1	Ratio of built-up area to urban administrative area	450	32.53	14.253	7.50	70.98
Land2	Green coverage rate of urban built-up areas	450	39.35	3.990	25.90	49.80
Land3	*Per Capita* area of paved roads	450	15.71	5.084	4.04	28.00
Soc1	Number of public transport vehicles in standard units per 10,000 population	450	12.34	3.125	6.83	26.55
Soc2	Urban water supply coverage rate	450	97.42	3.100	82.03	100.00
Soc3	Proportion of government health expenditure in total fiscal expenditure	450	7.61	1.637	3.90	13.93
Soc4	Number of urban assistant practicing physicians per 10,000 population	450	40.84	7.345	22.29	60.01
Soc5	Number of healthcare institution beds per 10,000 population	450	51.33	14.059	23.11	84.32

### 2.2 Random forest

The Random Forest algorithm, proposed by Breiman (30), is an ensemble learning-based method that extends the Bagging algorithm. It trains a decision tree on each subset and randomly selects a subset of features for splitting at each node, increasing feature randomness and reducing correlations between decision trees. The prediction results are obtained by averaging or voting the predictions of all trees. This process helps to reduce variance and errors, preventing both overfitting and underfitting. Each feature is ranked according to its assigned weight, referred to as feature importance (31). Through multiple random sampling and feature selection, Random Forest effectively handles high-dimensional data and uses tree-based ensemble methods to capture complex non-linear relationships between variables. Random forests evaluate feature contributions by calculating the Gini index after each feature split. Feature importance is defined as the total reduction in Gini impurity achieved by all splits involving that feature across the ensemble of trees. A higher value indicates a greater contribution of the feature to the classification task.

### 2.3 Gradient Boosting Decision Tree (GBDT)

Gradient Boosting Decision Tree (GBDT) is a Boosting algorithm with CART regression trees as the base learners, primarily aimed at optimizing general loss functions. The core idea is to fit the residuals of the previous round’s base learner using the negative gradient of the loss function, progressively reducing the residuals with each round. This approach ensures that the output of each round’s base learner approaches the true values. By fitting along the negative gradient direction, the method guarantees that the loss function decreases as rapidly as possible in each training round, accelerating convergence to a local or global optimal solution (29). GBDT is a powerful ensemble learning method capable of handling both continuous and discrete values. By iteratively optimizing the loss function, it captures complex nonlinear relationships, and the stage-by-stage residual fitting process ensures robustness even in the presence of noise, with regularization parameters effectively preventing overfitting. The calculation of feature importance in GBDT essentially quantifies “the aggregate contribution of a feature to the reduction of the loss function across all trees.” The more frequently a feature is utilized for node splitting, and the more significantly each split reduces the loss function, the higher its importance weight.

### 2.4 XGBoost

XGBoost is an efficient implementation of GBDT, which first uses the training set and sample true values to train a tree. This tree is then used to predict the training set, and the residuals (i.e., the differences between the predicted and true values) are calculated. The residuals are then used as the target for training the second tree. After the second tree is trained, the residuals are recalculated for each sample, and a third tree is trained, and so on (32). XGBoost utilizes a second-order Taylor expansion to more accurately approximate the optimal solution of the loss function, thereby improving prediction accuracy. During decision tree construction, it uses an “approximate greedy algorithm” to reduce computational complexity. The method also supports custom loss functions and evaluation metrics, allowing model parameters to be adjusted according to the research problem. Additionally, it can handle missing values automatically and supports parallel computation. XGBoost quantifies feature importance by measuring the reduction in the objective function (e.g., loss function) achieved at each split, referred to as the “gain.” After each split, the gain score of feature A accumulates the reduction in loss attributable to that split. The final importance score for feature A is obtained by summing its total gains across all trees. A higher gain indicates that the feature plays a more significant role in distinguishing samples.

### 2.5 Performance evaluation

The Root Mean Squared Error (RMSE), Mean Absolute Percentage Error (MAPE), and Mean Absolute Error (MAE) are the main metrics for evaluating the model’s prediction performance. The coefficient of determination (*R*^2^) is used to assess the goodness of fit of the model, and a higher *R*^2^ value indicates a superior goodness of fit. MAE represents the average absolute error between predicted and actual values, MAPE indicates the degree of data dispersion, and RMSE measures both the error between actual and predicted values and the dispersion between the two errors. The smaller these metrics, the better the model’s performance. However, all three metrics are influenced by sample values and predicted values, and there is no predefined standard. Given that K-fold cross-validation is appropriate for small datasets and facilitates effective use of all data for training and evaluation, and to ensure that each fold contains an adequate number of samples during cross-validation while preventing sample structure imbalance, this study adopts three-fold and four-fold cross-validation. The evaluation is conducted using holdout samples under various training/testing splits (70, 80, and 90%). For Boosting-based models (GBDT, XGBoost), their mechanism of sequentially fitting residuals tends to drive training error toward 0 and training *R*^2^ toward 1 when the number of iterations increases. Since RMSE has a broader evaluation range, this study chooses *R*^2^ and RMSE to evaluate prediction performance.

### 2.6 Software implementation

In this study, SPSSPRO is used to execute all algorithms, employing machine learning regression modules such as Random Forest, GBDT, and XGBoost. Table 1 shows the descriptive statistical characteristics of variables.

## 3 Results

Prior to modeling, a correlation analysis is conducted across all variables. While the correlation coefficient between ‘Ratio of Built-up Area to Urban Administrative Area’ and ‘Urban Population Density’ exceeds 0.8, all other inter-variable correlations remain below 0.7. Given that these two variables capture distinct aspects—the role of land use and population dynamics in urbanization—they are retained in the model. All machine learning methods employed in this study incorporated data shuffling and cross-validation procedures. Using random forest as the baseline model, parameter tuning and methodological adjustments are systematically implemented to identify robust computational outcomes.

### 3.1 Random forest results

Initial implementation allocates 70% of samples to the training set and 30% to the test set, with three-fold cross-validation. As Table 2 shows, the results indicate a training set RMSE of 27.454 and a test set RMSE of 48.524, corresponding to coefficients of determination (*R*^2^) of 0.93 and 0.696, respectively.

**Table 2:** Comparison of predictive power for different training set shares.

Set	70% training set share		80% training set share		90% training set share	
	RMSE	*R* ^2^	RMSE	*R* ^2^	RMSE	*R* ^2^
Training set	27.454	0.93	24.796	0.937	25.113	0.938
Test set	48.542	0.696	54.869	0.708	41.859	0.773

Subsequent trials adjusted the training set proportions to 80 and 90%. While test set RMSE decreased with larger training sets, the 80% training allocation exhibits lower training set RMSE (23.117) compared to the 90% partition (25.832). However, the 90% training configuration demonstrates superior generalization performance, achieving a higher *R*^2^. Comparative analysis of these metrics suggests the 90% training ratio optimizes predictive accuracy and model fit.

To mitigate potential overfitting risks inherent in high training set allocations (90%), this study adopts complementary methodologies to enhance model generalizability and investigates the model’s adaptability to varying data volumes. Subsequent sections detail these refinements and their validation outcomes.

### 3.2 Comparison of three algorithms

Table 3 presents the performance metrics of three algorithms under varying training ratios and cross-validation folds.

**Table 3:** Comparison of predictive ability of different algorithms.

Set and Scenario	Random Forest		GBDT		XGBoost	
	RMSE	*R* ^2^	RMSE	*R* ^2^	RMSE	*R* ^2^
Scenario 1: 70% training set share, 3 folds						
Training set	27.454	0.93	0.124	1	0.573	1
Test set	48.542	0.696	47.721	0.754	51.397	0.735
Scenario 2: 80% training set share, 3 folds						
Training Set	24.796	0.937	0.216	1	0.406	1
Test set	54.869	0.708	44.278	0.78	62.001	0.371
Scenario 3: 70% training set share, 4 folds						
Training set	24.402	0.94	0.224	1	0.386	1
Test set	52.471	0.718	45.725	0.778	45.741	0.774
Scenario 4: 80% training set share, 4 folds						
Training set	24.742	0.941	0.252	1	0.543	1
Test set	52.564	0.662	45.734	0.829	36.48	0.772

In Scenario 1, the GBDT algorithm achieved the lowest RMSE values (training set: 0.124; test set: 47.721), significantly outperforming the other two algorithms. Correspondingly, its coefficients of determination (*R*^2^) for both training and test sets surpasses those of alternative methods. Similar trends are observed in Scenario 2. However, in Scenario 3, GBDT and XGBoost demonstrated comparable performance without statistically significant differences in metrics. In Scenario 4, GBDT exhibits a lower test set RMSE than XGBoost, while achieving the highest *R*^2^ value.

Six optimal configurations are subsequently identified: GBDT (Scenario 1), GBDT (Scenario 2), GBDT/XGBoost (Scenario 3), and GBDT/XGBoost (Scenario 4). Comparative analysis reveals that GBDT (Scenario 3) delivers the lowest RMSE values across both training and test sets. Although its *R*^2^ (0.783) marginally trails that of GBDT (Scenario 4, *R*^2^ = 0.812), the negligible performance gap (<3%) prioritizes RMSE minimization for model selection. Consequently, GBDT (Scenario 3) is deemed the optimal predictor, followed by GBDT (Scenario 4) and XGBoost (Scenario 3). The boosting models achieve near-perfect fits on the training data (*R*^2^ = 1) while exhibiting a generalization gap on the test sets—a common pattern under noisy observational data. The selected specification best balances bias and variance, yielding the lowest test RMSE. Subsequent analyses focuses on these three configurations.

### 3.3 Variable importance ranking

Table 4 summarizes variable importance rankings across three selected configurations. Social factors consistently dominated all models, accounting for over 45% of total feature weights, with GBDT (Scenario 4) reaching 0.477.

**Table 4:** Ranking of the importance of each type of factor.

Factor	GBDT (Scenario 3)	GBDT (Scenario 4)	XGBoost (Scenario 3)	Average results
Society	0.457	0.477	0.467	0.467
Land	0.115	0.229	0.301	0.215
Population	0.282	0.156	0.074	0.17
Economy	0.147	0.139	0.158	0.148

**Figure 2:**
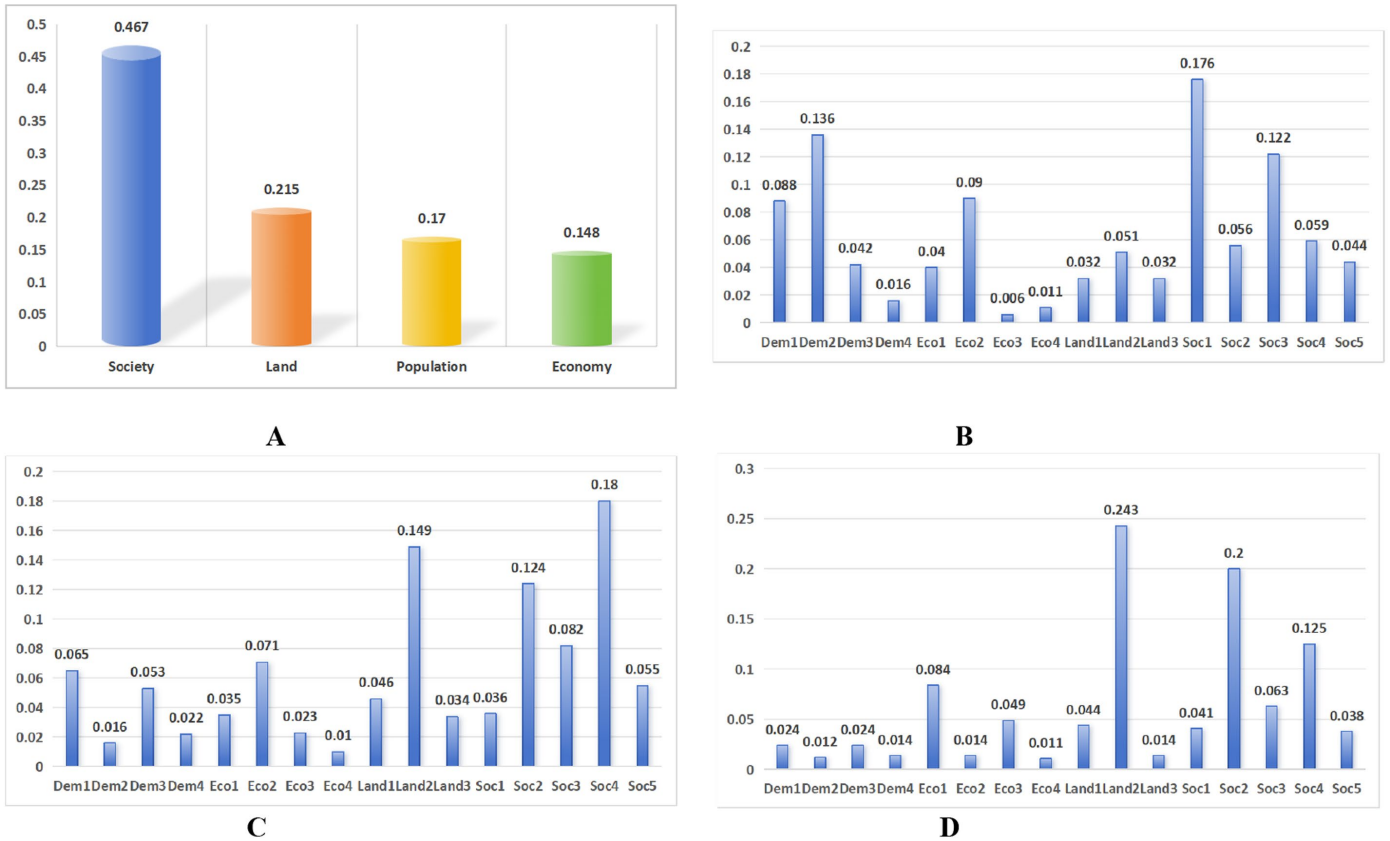
Ranking of the weights of the various elements and variables. **(A)** Average results. **(B)** GBDT (scenario 3). **(C)** GBDT (scenario 4). **(D)** XGBoost (scenario 3).

Discrepancies emerged among secondary factors: GBDT (Scenario 3) prioritized demographic factors (28.1%), followed by economic (15.6%) and land-related (11.3%) elements, whereas GBDT (Scenario 4) and XGBoost (Scenario 3) assigned higher weights to land factors (22.4 and 19.8%, respectively), relegating demographic and economic factors to tertiary positions. A composite ranking derived from mean importance scores established the hierarchy as: social factors > land factors > demographic factors > economic factors. Quantitative weight calculations are shown in Table 5 and Figure 2.

**Table 5:** Results of variable weight calculation.

Type	Variables	GBDT (Scenario 3)	GBDT (Scenario 4)	XGBoost (Scenario 3)
Dem1	Total dependency ratio	0.088	0.065	0.024
Dem2	Urbanization rate	0.136	0.016	0.012
Dem3	Illiteracy rate	0.042	0.053	0.024
Dem4	Urban population density	0.016	0.022	0.014
Eco1	Ratio of tertiary industry value-added to secondary industry value-added	0.04	0.035	0.084
Eco2	Foreign trade dependence	0.09	0.071	0.014
Eco3	*Per Capita* disposable income of urban residents	0.006	0.023	0.049
Eco4	Ratio of completed investment in industrial pollution control to GDP	0.011	0.01	0.011
Land1	Ratio of built-up area to urban administrative area	0.032	0.046	0.044
Land2	Green coverage rate of urban built-up areas	0.051	0.149	0.243
Land3	*Per Capita* area of paved roads	0.032	0.034	0.014
Soc1	Number of public transport vehicles in standard units per 10,000 population	0.176	0.036	0.041
Soc2	Urban water supply coverage rate	0.056	0.124	0.2
Soc3	Proportion of government health expenditure in total fiscal expenditure	0.122	0.082	0.063
Soc4	Number of urban assistant practicing physicians per 10,000 population	0.059	0.180	0.125
Soc5	Number of healthcare institution beds per 10,000 population	0.044	0.055	0.038

### 3.4 Grouped sample analysis

China spans a vast territory with significant geographical diversity. There are notable differences across provinces in terms of geographical location, resource endowment, industrial structure, and degree of openness. Additionally, the severe imbalance in regional development leads to regional variations in the impact of rapid urbanization on public health. Therefore, in this study, the 30 provincial-level units of Mainland China are divided into three regions, as outlined in Table 6.

**Table 6:** Provinces included in each region.

Region	Provinces
Eastern	Beijing, Tianjin, Hebei, Liaoning, Shandong, Shanghai, Jiangsu, Zhejiang, Fujian, Guangdong, Hainan
Central	Shanxi, Jilin, Heilongjiang, Anhui, Jiangxi, Henan, Hubei, Hunan
Western	Inner Mongolia, Guangxi, Chongqing, Sichuan, Guizhou, Yunnan, Shaanxi, Gansu, Ningxia, Qinghai, Xinjiang

Given that the GBDT algorithm outperforms other algorithms, this section continues to use the GBDT algorithm to discuss the situation in different regions. The training set accounts for 70%, and a four-fold cross-validation method is similarly employed. The predictive performance is shown in Table 7.

**Table 7:** Model forecasting capabilities on the three regions.

Set	Eastern		Central		Western	
	RMSE	*R* ^2^	RMSE	*R* ^2^	RMSE	*R* ^2^
Training Set	0.003	1	0.002	1	0.005	1
Test Set	36.514	0.805	29.824	0.628	47.296	0.847

Table 7 shows that there is no significant difference in the RMSE of the training sets across the three regions. In the test set, the western region has the highest RMSE, followed by the eastern region, while the central region exhibits the lowest RMSE. The *R*^2^ values of the training sets for all three regions are satisfactory. However, in the test set, the *R*^2^ for the central region is less, which may be attributed to the relatively smaller sample size in the central region.

**Figure 3:**
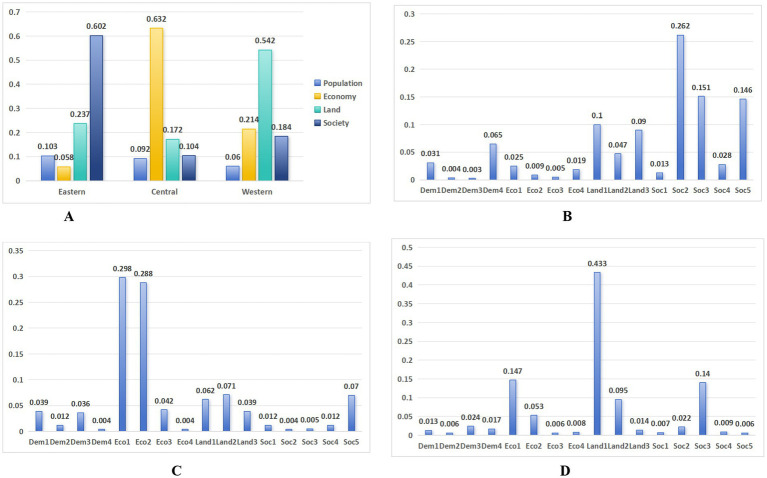
Ranking of the weights of all elements and variables in the three regions. **(A)** Weighting of elements in the three regions. **(B)** Eastern. **(C)** Central. **(D)** Western.

Table 8 presents the weights of various factors across the three major regions. In the eastern region, the most significant factor is the social factor, with a weight of 60%, followed by land factors, which account for nearly a quarter, and the lowest is the economic factor, with a weight just slightly above 5%. In the central region, economic factors dominate, with a weight exceeding 60%, followed by land factors, which account for 17.2%, while population factors have the lowest weight, under 10%. In the western region, land factors take the lead, with a weight greater than half, followed by economic factors, which account for more than one-fifth, and population factors also represent a relatively small proportion. Table 9 and Figure 3 show the weight calculation results for the variables in the three regions, which also exhibit considerable differences.

**Table 8:** Weights of factors in the three regions.

Factor	Eastern	Central	Western
Population	0.103	0.092	0.06
Economy	0.058	0.632	0.214
Land	0.237	0.172	0.542
Society	0.602	0.104	0.184

**Table 9:** The weights of the variables in the three regions.

Type	Variables	Eastern	Central	Western
Dem1	Total dependency ratio	0.031	0.039	0.013
Dem2	Urbanization rate	0.004	0.012	0.006
Dem3	Illiteracy rate	0.003	0.036	0.024
Dem4	Urban population density	0.065	0.004	0.017
Eco1	Ratio of tertiary industry value-added to secondary industry value-added	0.025	0.298	0.147
Eco2	Foreign trade dependence	0.009	0.288	0.053
Eco3	*Per Capita* disposable income of urban residents	0.005	0.042	0.006
Eco4	Ratio of completed investment in industrial pollution control to GDP	0.019	0.004	0.008
Land1	Ratio of built-up area to urban administrative area	0.100	0.062	0.433
Land2	Green coverage rate of urban built-up areas	0.047	0.071	0.095
Land3	*Per Capita* area of paved roads	0.090	0.039	0.014
Soc1	Number of public transport vehicles in standard units per 10,000 population	0.013	0.012	0.007
Soc2	Urban water supply coverage rate	0.262	0.004	0.022
Soc3	Proportion of government health expenditure in total fiscal expenditure	0.151	0.005	0.140
Soc4	Number of urban assistant practicing physicians per 10,000 population	0.028	0.012	0.009
Soc5	Number of healthcare institution beds per 10,000 population	0.146	0.070	0.006

## 4 Discussion

In a narrow sense, urbanization refers to the process of population concentration from rural to urban areas. In a broader sense, urbanization is often accompanied by profound changes in the economy, land use, and social structure. It is not only an economic development process but also a comprehensive manifestation of population migration, land use transformation, and social change. From a demographic perspective, urbanization is characterized by a rapid increase in the proportion of urban population relative to the total population, which serves as the primary statistical indicator for measuring the level of urbanization. Economically, urbanization drives the transformation of industrial structure, with labor shifting from agriculture-based primary industries to industry-dominated secondary industries and service-oriented tertiary industries. Industrialization plays a crucial role as a driving force for urbanization. In terms of land use, urbanization leads to changes in land utilization, with agricultural land being converted into urban construction land, thereby expanding urban spatial boundaries. At the social level, urbanization also alters the living environment, such as improvements in infrastructure and the widespread availability of public services. However, while urbanization improves residents’ well-being, it also gives rise to new social issues, such as high population density, ecological degradation, and deteriorating living conditions. This complexity and diversity of urbanization inevitably result in a multifaceted impact on infectious disease prevention and control. The influence of rapid urbanization on public health has evolved from a singular risk of population aggregation to a complex system of interactions involving environmental capacity, economic transformation, and resource allocation. Based on the weight ranking of different algorithms discussed in the previous section, the following main conclusions can be drawn.

### 4.1 Social factors as primary determinants

Social factors, with a weight of 0.467, rank first among all influencing factors, indicating their predominant influence on public health throughout urbanization. Social factors include healthcare and sanitation expenditures, public health facilities, and the accessibility of urban public transportation, reflecting the crucial role of infrastructure in the rapid urbanization process. Fitch (33) emphasized that well-developed infrastructure is essential for preventing and controlling epidemics, as it directly affects residents’ access to healthcare resources and their capacity to cope with diseases. Indicators such as the proportion of healthcare spending in general fiscal expenditure, the number of urban practicing assistant physicians per 10,000 people, and the number of hospital beds per 10,000 people reflect the distribution of public health services during urbanization. If public health service provision can keep pace with urbanization, it can effectively alleviate the supply–demand imbalance in healthcare and control the scope of infectious diseases. The medical resource shortages during the COVID-19 pandemic highlighted that higher healthcare investment, more specialized physicians, and broader disease prevention programs are crucial for controlling the spread of epidemics. Indicators such as the number of public transportation vehicles per 10,000 people and urban water supply coverage reflect the convenience of urban living conditions. A well-developed public transportation system can reduce the time cost for residents to access healthcare services, enhance the accessibility of medical services, and decrease the use of private cars, thereby reducing traffic congestion and emissions, and improving air quality. However, public transportation vehicles are crowded public spaces and enclosed environments, which inevitably become breeding grounds for the transmission of infectious diseases. They can also facilitate the spread of viruses from one area to another, contributing to the dissemination of diseases (34). Safe drinking water supply is fundamental for preventing diseases such as enteric infections (35). The 1854 cholera outbreak on Broad Street in London, during the first industrial revolution, was primarily caused by contaminated drinking water. Therefore, during urbanization, the government must prioritize the construction and improvement of the social service system. By increasing healthcare investment, optimizing public transportation infrastructure, and improving water supply coverage, the urban response capacity to public health events can be enhanced, ensuring that prevention and control measures are implemented quickly and effectively, thus controlling the spread and speed of epidemics.

### 4.2 Land factors as structural influences

Land factors rank second in significance, with an average weight of 0.215, underscoring their essential role in shaping infectious disease patterns. Rapid urban growth intensifies land conversion and population agglomeration, rendering prudent spatial planning indispensable. Poorly designed land-use systems—particularly those characterized by unbalanced development or inadequate green infrastructure—tend to give rise to overcrowded settlements and unsanitary conditions conducive to disease transmission (36). The green coverage rate in built-up areas occupies the largest proportion among land-related indicators, suggesting that a higher green coverage rate not only improves air quality but also absorbs airborne pathogens, thereby reducing the likelihood of residents contracting diseases (8, 23, 37). Per capita road area reflects the smoothness of traffic flow, and smooth transportation is beneficial for the rapid passage of emergency medical vehicles, significantly shortening emergency response times and improving the efficiency of medical rescue efforts. However, it can also serve as an important medium for the spread of viruses, providing a rapid channel for viral transmission. This contradiction stems from the dual nature of the transportation system in terms of spatial mobility and social connectivity. Moreover, indicators such as the proportion of built-up areas relative to total land and per capita urban road area generally remain low in direct correlations with infection incidence, likely because these parameters influence health through longer, indirect feedback loops rather than immediate effects. Consequently, sustainable urban planning should conform to ecological principles by balancing expansion with environmental preservation. Expanding green zones, regulating urban density, and designing efficient road networks collectively enhance urban livability while constraining the spatial scope of disease diffusion. For most developing countries, particular attention should be paid to the sustainable development of suburban areas, and systemic measures should be implemented to eliminate potential sources of infection.

### 4.3 Population factors as potential risks

The average weight of population factors is 0.17, exert measurable yet less direct effects compared to social and land determinants. Their influence derives primarily from the contingent risks embedded in population distribution and demographic composition. The total dependency ratio, an indicator of age structure, illustrates the socioeconomic burdens related to non-working populations. Elevated dependency ratios correspond to a higher proportion of older adults and juvenile residents—groups inherently more susceptible to infectious diseases due to weaker immune capacity. A disproportionate presence of such vulnerable demographics intensifies the likelihood of disease spread. The proportion of urban population is an important indicator of urbanization. In the short term, the large-scale concentration of population in cities increases the frequency of person-to-person contact, accelerating the spread of infectious diseases and expanding their transmission range. If health infrastructure lags behind the speed of urbanization, it also adds pressure to the operation of existing urban infrastructure, worsening living conditions. For example, densely populated areas such as urban villages, where sanitation facilities are relatively underdeveloped, can easily become sources of infection (38). However, population concentration can also facilitate the centralized provision of public health services, improving the efficiency of medical resource utilization. Illiteracy rates reflect the quality of the population. Residents with higher levels of education are generally better able to access and absorb knowledge related to infectious disease prevention and control, applying it in their daily lives, and are more likely to proactively share such knowledge. Therefore, governments should pay close attention to changes in population structure during the urbanization process, strengthen the management and health education of the mobile population, widely disseminate public health knowledge, and effectively protect the rights of vulnerable groups while ensuring the implementation of basic healthcare protection.

### 4.4 Economic factors as underlying conditions

The average weight of economic factors is 0.148, possess the least direct impact on infectious disease prevalence, yet they underpin the broader environment that determines public health capacity. Economic growth elevates material living conditions and provides fiscal resources for healthcare expansion; however, without deliberate prioritization of public health infrastructure, infection rates may persist despite increased wealth. From the perspective of different indicators, the ratio of the value added by the tertiary industry to that of the secondary industry is an important measure of the optimization and upgrading of a country’s or region’s industrial structure. An increase in the share of the tertiary industry typically indicates a shift toward a service-oriented economy, accompanied by an improved production environment and a relative reduction in occupational hazards. For example, compared to traditional manufacturing, high-tech industries demonstrate better resource utilization efficiency and environmental friendliness. Certain sectors of the tertiary industry, closely related to residents’ health—such as pharmaceutical research and development, healthcare services, and professional technical services—directly reinforce societal health by promoting longevity and expanding access to scientific advancements. These sectors generally have high knowledge intensity and technical added value, effectively promoting high-quality employment, facilitating the transformation and application of scientific and technological achievements, and driving the continuous optimization and upgrading of the economic structure. This, in turn, provides solid support for the realization of the “Healthy China” strategic goals. The ratio of total imports and exports to GDP reflects the degree of openness. In the context of globalization, urbanization is closely linked to international trade. A higher share of imports and exports indicates frequent exchanges between cities and the outside world, which could bring advanced medical technologies but also increases the risk of cross-border transmission of infectious diseases. From July to August 2020, eight cases of COVID-19 were detected on the packaging of imported cold-chain products in Liaoning, Fujian, Jiangxi, Chongqing, Yunnan, Shandong, and Anhui, highlighting that infectious viruses can be transmitted to cities through international business travel and goods transportation (39). The per capita disposable income of urban residents influences their capacity for health-related consumption. As income rises, residents are more willing and able to undergo regular check-ups, vaccinations, and early treatment, enabling “early detection, early diagnosis, and early treatment,” and affording better medical services and medications. The proportion of investment in industrial pollution control relative to GDP reflects the extent of industrial pollution management. Insufficient investment in this area could result in industrial pollution damaging residents’ respiratory and digestive systems, thereby increasing the incidence of infectious diseases. For example, six out of the eight major pollution incidents that occurred during the golden age of capitalism between the 1950s and 1960s were linked to industrial pollution. Although the weight of economic factors is low, they still form the material foundation for the establishment of public health and medical systems. Therefore, during the urbanization process, governments need to focus on coordinating economic development and environmental protection. By increasing investments in industrial pollution control, promoting industrial structure upgrading, and raising residents’ income levels, a solid economic foundation for public health can be created. For instance, the government could encourage businesses to adopt environmentally friendly production technologies and develop green industries to reduce the impact of industrial pollution on residents’ health.

### 4.5 Regional differentiation in health impacts

In the eastern region, social factors dominate, signifying that public health outcomes rely heavily on efficient allocation of public service resources. This aligns with the characteristics of the mature urbanization stage in the east, where high population concentration forces the optimization of medical resources and infrastructure. This is reflected in the proportion of health expenditure in total fiscal expenditure and the number of healthcare institution beds per 10,000 population, both around 0.15, while urban water supply coverage rate reaches 0.262. Equalization of public services can effectively break the transmission chain of infectious diseases.

In the central region, economic factors exert greater weight, exposing structural tensions characteristic of mid-industrialization phases. The weight of industrial structure transformation indicators (ratio of tertiary industry value-added to secondary industry value-added) and openness indicators (ratio of foreign trade dependence) approaches 30%, driving the risk of infectious diseases. This corresponds to the “Environmental Kuznets Curve” theory—during industrial transfer in the central region, lagging environmental regulations increase occupational exposure risks, making it more likely for infectious disease outbreaks.

In contrast, the western region is defined primarily by land-based determinants. The ecological environment in the west is relatively fragile, and excessive urban expansion may damage the original vegetation and soil structure, leading to desertification, soil erosion, and more dust storms. Dust carries bacteria, viruses, and other pathogens, which can increase the incidence of respiratory infectious diseases. Some western regions are natural plague foci, where wild animals serve as hosts or vectors for infectious diseases. When their habitats are disturbed, increased contact with humans can raise the risk of zoonotic disease transmission (38). The eastern region should provide high-quality medical and health resources and improve the public health security system. The central region should alleviate the contradiction between the economy and the environment during the process of industrialization and pursue green and sustainable development. The western region should attach importance to the protection of natural ecology and curb the spread of zoonotic diseases.

We explicitly acknowledge slight overfitting signals in the training phase and underfitting on the test sets. The gap mainly reflects (i) small-sample, high-heterogeneity provincial panels, (ii) unobserved spatial spillovers and interactions not modeled her, and (iii) structural breaks during rapid urbanization and the COVID-19 period. Our design mitigates—but cannot eliminate—these risks via cross-validation, repeated shuffling, and prioritizing out-of-sample metrics; thus, results should be interpreted with caution.

## 5 Conclusion

This study employs machine learning algorithms including Random Forest, GBDT, and XGBoost to investigate the impacts of rapid urbanization on public health, which holds significant theoretical and practical significance. Theoretically, it clarifies the hierarchical order of the influences of social, land, demographic, economic and other factors on public health, reveals the action mechanisms of each factor from multiple dimensions, and enriches the theoretical system in this field. The findings reveal a hierarchical order of influential factors: social factors emerge as the most critical determinant, followed by land-related factors, demographic factors, and economic factors. Additionally, it identifies the spatial heterogeneity in the interaction between urbanization and health, providing a new perspective for understanding how regional urbanization characteristics and ecological constraints affect public health. Practically, it offers a scientific basis for governments to formulate urbanization and public health policies, facilitates the implementation of differentiated strategies based on the dominant factors in different regions, and helps prevent and control public health risks arising from population structure changes, economic activities and other aspects in advance.

Regarding social dimensions, public transportation accessibility, water supply coverage, healthcare investment, and medical resource allocation directly influence population health outcomes and the accessibility of public health services. In the land-use domain, rational planning of built-up areas, green space coverage, and road infrastructure demonstrate significant health-protective effects, whereas improper land utilization may induce systemic health risks. Demographic analysis indicates that population structure shifts, spatial agglomeration patterns, and educational attainment levels present dual effects, creating opportunities for centralized public health service delivery while simultaneously amplifying infectious disease transmission risks. Economic factors exhibit paradoxical impacts: while industrial pollution control, industrial upgrading, international trade, and income growth contribute positively to public health, they concurrently harbor potential threats such as environmental degradation and cross-border transmission of infectious diseases.

Notably, spatial heterogeneity manifests in urbanization-health interactions across regions: social factors dominate in eastern China, economic factors prevail in central China, and land-related factors exert paramount influence in western China. This geographical stratification reflects the differential developmental priorities and ecological constraints inherent to China’s regional urbanization trajectories.

### 5.1 Limitation

This study has several limitations. Due to methodological constraints, we did not account for spatial spillover effects among provincial units or interactions between different factors. Future research could be further advanced in multiple dimensions. On the one hand, it is necessary to incorporate the spatial spillover effects among provincial administrative units and the interaction effects of different factors. By constructing a more comprehensive model, this study can accurately capture inter-regional mutual influences and the combined effects of factors, thereby providing precise policy recommendations for regional coordinated development. On the other hand, further investigate the nonlinear relationships between specific variables and public health outcomes, and conduct long-term follow-up studies to observe the changing trends of impacts and long-term cumulative effects. Meanwhile, expand the research scope and sample size, integrate multidisciplinary approaches, and comprehensively apply theories and technologies from different disciplines to conduct in-depth research from multiple perspectives, so as to obtain more comprehensive and in-depth findings.

## Data Availability

The original contributions presented in the study are included in the article/supplementary material, further inquiries can be directed to the corresponding author.
